# Hearing Loss Characteristics of Workers with Hypertension Exposed to Occupational Noise: A Cross-Sectional Study of 270,033 Participants

**DOI:** 10.1155/2018/8541638

**Published:** 2018-12-19

**Authors:** Boshen Wang, Lei Han, Simin Dai, Xiuting Li, Wenyan Cai, Dandan Yang, Lin Chen, Ning Wang, Baoli Zhu, Juan Zhang

**Affiliations:** ^1^Key Laboratory of Environmental Medicine Engineering of Ministry of Education, School of Public Health, Southeast University, Nanjing 210009, Jiangsu, China; ^2^Jiangsu Provincial Center for Disease Prevention and Control, Nanjing 210009, Jiangsu, China; ^3^National Institute of Parasitic Diseases, Chinese Center for Disease Control and Prevention, China; ^4^Nanjing Prevention and Treatment Center for Occupational Diseases, Nanjing 210042, Jiangsu, China; ^5^Center for Global Health, School of Public Health, Nanjing Medical University, Nanjing 210000, Jiangsu, China

## Abstract

**Objectives:**

This study investigated the hearing loss characteristics among occupational noise exposure workers with hypertension and the link between hypertension and hearing loss when exposed to occupational noise.

**Methods:**

A total of 267,766 occupational noise-exposed workers were enrolled, including 29,868 workers with hypertension and 240,165 without hypertension. Hypertension was diagnosed according to WHO criteria. Hypertension was classified into four grades based on blood pressure. Assessment of hearing was performed through measurement of an unadulterated tone threshold at different frequencies, which ranged between 250 and 8,000 Hz.

**Results:**

A substantial link was observed to exist between hypertension and the increment in the hearing limit. The increase in the hearing threshold was substantially higher among those having grade 2 hypertension.

**Conclusion:**

The current investigation suggested patients with hypertension exhibit a substantial rise in hearing loss in comparison with patients without hypertension. The rise in hearing loss was significant in patients with grade 2 hypertension. Efficient and practicable measures are required to decrease the hearing loss in workers with hypertension and work-related noise exposure.

## 1. Introduction

Hearing loss (HL) is regarded as an element that influences the quality of life irrespective of the commitment level. As adults acquire the same, HL exhibits gradual appearance and might give rise to spoken language issues [1, 2].

Noise is the most important environmental factor and the most frequent occupational hazard, predominantly found among industrial workers [3]. According to information originating from the American Speech Language Hearing Association (ASHA) [4], 28 million persons in the US have various kinds of HL, of whom 80% cannot be reversed. In addition, the data illustrates that 4.6% of persons 18-44 years of age face hearing loss. In contrast, 14% of persons 45-64 years of age and 54% of persons > 65 years of age encounter HL because of various elements, such as severe and/or continued noise contact, breathing poisonous materials, absorption of ototoxic medicines and contaminants, injuries, and genetic inheritance.

Arterial hypertension is associated with significant morbidity and mortality [5]. Arterial hypertension is related to stroke, heart disease, kidney disease, and vascular disease [6]. To maintain proper function, every living cell is dependent on an adequate provision of both oxygen and nutrients. Moreover, delivery of oxygen and nutrients is dependent on functional, as well as structural veracity of the heart and blood vessels [7]. Hypertension, which is considered to be the most common vascular syndrome, is likely to extend structural modifications in the heart, as well as blood vessels [8]. In addition, elevated pressure in the vascular system is most likely to give rise to internal ear bleeding via the anterior inferior cerebellar artery, which extends support to the internal ear artery, and, together with the cochlear and anterior vestibular arteries, is likely to result in gradual or abrupt HL. This circulatory system pathology is likely to exert direct impact on hearing function in a number of ways [9]. Among the vascular physiopathologic phenomena are the rise in blood viscosity, decreased capillary blood flow, and reduction in oxygen transport, thus giving rise to tissue hypoxia and leading to auditory dysfunction as well as HL [10]. Furthermore, arterial hypertension is likely to lead to ionic modifications in cell capacities, thus giving rise to auditory loss [11].

The purpose of the current study was to determine the link between hypertension and HL. The outcomes obtained from the current investigation are expected to encourage more cooperation among otorhinolaryngologists, speech therapists, and other medical experts associated with auditory loss treatment, for the purpose of enhancing the standard of treatment and recovery in HL patients.

## 2. Methods

### 2.1. Subjects

The subjects in this investigation were 267,766 employees who were exposed to work-related noise. The recruitment of the subjects was carried out from the key occupational-disease monitoring information system of the work-related noise coverage in Jiangsu Province, China. In the key occupational-disease monitoring information system, the subjects were employees recruited in noise-prone manufacturing units of mechanical tools, domestic appliance production, steel construction, and cigarette manufacture/packaging in Jiangsu Province, China. The workers who wore auditory aids or had drug-induced deafness were excluded from the study. The subjects faced work-related noise for a period > 1 year where the noise exposure had an intensity > 80 dB (A) (LEX, 8h). Determination of the intensity of noise in the working environment was performed with the help of a noise statistical analyser (AWA6218; Westernization Instrument Technology Co., Ltd., Beijing, China). Evaluation of the noise exposure was carried out using the equivalent continuous dB (A) weighted sound pressure levels (LEX, 8h), as recommended by the Occupational Health Standard of the People's Republic of China: Measurement of Noise in the Workplace [GBZ/T 189.8–2007] (China, 2007).

### 2.2. Blood Pressure Measurement and Definition of Hypertension

As per the standard protocol, subsequent to > 12h of noise contact, recording of the systolic blood pressure (SBP) and diastolic blood pressure (DBP) was carried out by trained physicians. The measurement was performed with the use of a mercury sphygmomanometer with the subjects sitting following ≥ 15 min of rest. Reporting the SBP and DBP made the average of 4 recurring calculations using 30 sec intervals. The definition of hypertension was a SBP ≥ 140 mmHg and/or a DBP ≥ 90 mmHg. Analysis of the data included by the medical examination report was performed. Subjects were categorized by different levels of hypertension in accordance with the blood pressure recordings, as stated by the WHO with respect to hypertension. In general, the diagnosis of hypertension is carried out on the bases of a consistently elevated blood pressure. As evident from Table 1, the WHO/ISH blood pressure classification integrates four levels of hypertension.

### 2.3. Pure Tone Audiometry

An otolaryngologist inspected the ears of each subject who had undergone pure tone audiometry testing in a sound-adjusting cabin with a contextual noise degree <25 dB (A). Testing of both ears was carried out through the use of soaring pure tones at frequencies of 0.5, 1, 2, 3, 4, and 6 kHz. The subjects were required to avoid a loud atmosphere for > 12 h prior to the test. Repetitions of the trials were performed a minimum of 3 times for determination of the least signal severity, which served as the ultimate threshold value for all ears. The use of mean threshold values at 0.5, 1, and 2 kHz was made for determination of the low-frequency hearing position. In contrast, the mean threshold values at 3, 4, and 6 kHz were utilized for determination of the high-frequency hearing position. In addition, a nick of the noise-induced hearing loss (NIHL) was displayed at approximately 3-6 kHz. Moreover, the threshold values at high frequency were considerably worse compared with the threshold values at low frequency. Definition of the audiometric shortcoming or HL was put forward as a hearing threshold >25 dB, subjected to the high frequency or high and low frequencies. The diagnostic criteria of work-related NIHL were developed on the bases of the Chinese work-related health standards (GBZ49-2014, http://www.zybw.net). On a specific test frequency, the definition of normal hearing was designated as the binaural hearing level ≤25 dB. Furthermore, the definition of unilateral hearing loss was made as monaural hearing >25 dB. In addition, the bilateral hearing loss was termed binaural hearing level >25 dB.

### 2.4. Statistical Analysis

Ascertainment of the cumulative noise exposure (CNE) was performed as follows: CNE =10× log(10 SPL/10 ×years of noise exposure), wherein the SPL indicates the sound pressure level [dB (A)] of the noise contact. Expression of the continued variables for the normal distribution was the mean ± standard deviation (SD). Expression of the categorical variables was frequencies, for the purpose of analysing the statistical relevance of categorical variables (%). A *χ*2 test was used when indicated. One-way ANOVA was used for continuous variables, following several comparisons with the Student-Newman-Keuls test. The dissimilarities existing between the subjects with HL subsequent to the adjustment of confounders, such as age, gender, working age, work age of noise contact, SBP and DBP, and binary logistic regression, were compared. Ascertainment of the odds ratio (OR) and 95 percent confidence interval (CI) was made for the risk of hypertension through noise contact. Performance of statistical analysis was carried out using SPSS 20.0 for Windows (IBM Corporation, Armonk, NY, USA).

## 3. Results

An aggregate of 267,766 subjects were enrolled in our investigation, of whom 240,165 comprised the control subjects with no hypertension and 27,601 were diagnosed with hypertension. In addition, 12,723 patients were diagnosed with grade 1 hypertension in accordance with the WHO categorization for hypertension. Moreover, 1990, 585, and 12,303 patients were diagnosed with grade 2, 3, and 4 hypertension, respectively. Patients with grade 2 hypertension had the largest proportion of HL workers (*P*<0.05; Table 2).

**Figure 1:**
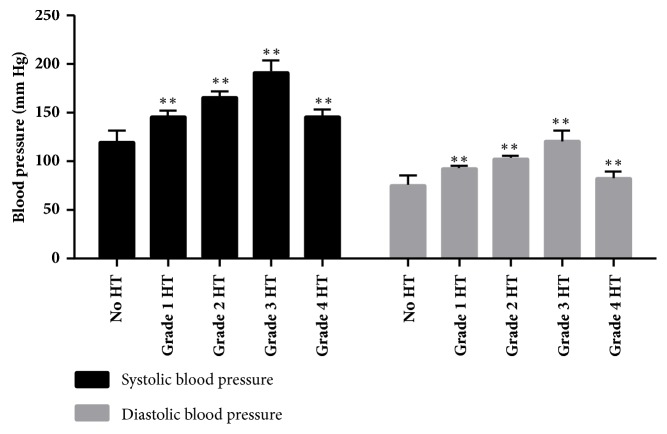
Blood pressure of the subjects with occupational noise exposure, ^*∗*^*P*<0.05, ^*∗∗*^*P *<0.01, in comparison with the No HT group.

The mean levels of SBP were as follows: 119.89 ±11.22 mmHg (No HT); 145.96±5.58 mmHg (grade 1 HT); 165.55±5.21 mmHg (grade 2 HT); 190.91±12.23 mmHg (grade 3 HT); and 145.29±6.74 mmHg (grade 4 HT). The mean levels of DBP were as follows: 76.04±8.74 mmHg (No HT); 93.27±3.04 mmHg (grade 1 HT); 103.40±3.05 mmHg (grade 2 HT); 120.66±10.00 mmHg (grade 3 HT); and 82.87±6.03 mmHg (grade 4 HT).  Figure 1 shows the percentages of subjects with hypertension, together with systolic hypertension and diastolic hypertension, which were substantially greater in the hypertension cohort in comparison with the No HT cohort (*P*<0.05).

**Figure 2:**
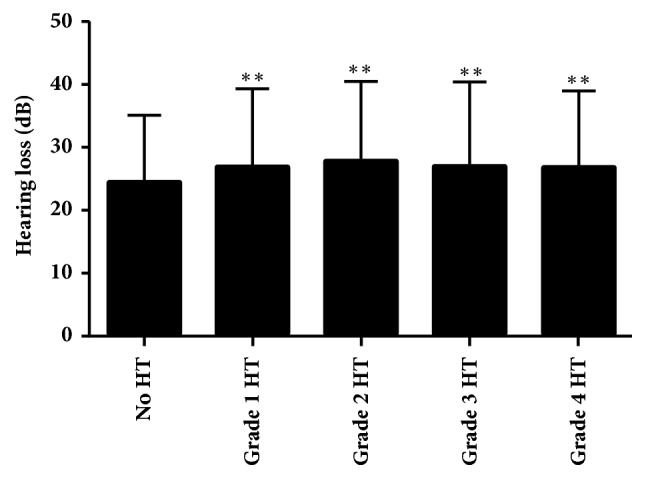
Mean pure tone thresholds by hypertension severity, ^*∗*^*P*<0.05, ^*∗∗*^*P*<0.01, in comparison with the No HT group.

Figure 2 provides the pure tone threshold outcomes of the two ears calculated in accordance with the levels of hypertension. Moreover, the rise in the hearing threshold was higher in the patients with grade 2 hypertension. The* p* value recorded with the use of the Pearson's correlation technique was < 0.01, which revealed a substantial link between hypertension and the rise in hearing limits.

The logistic regression analysis was employed for the purpose of accessing the association between simultaneous hypertension and hearing loss in a more precise manner, and additionally adjusting the confounding factors. Additionally, the independent variables integrated the age, gender, working age, work age of noise, and SBP and DBP. The outcomes of this investigation revealed that following adjustment of the confounding variables, there was a substantial association among concurrent HL and hypertension. In addition, the OR of the HL cohort in comparison with the No HL group, the subjects with SBP and DBP exhibited a significantly higher risk with ORs (95% CIs) of 1.046 (1.032-1.060) and 1.003 (1.001-1.005), respectively. Tables 3 and 4 show the occurrence of hypertension among the alternate workers in accordance with the hypertension risk elements and HL. On the bases of Tables 3 and 4, HL is likely to be augmented as a function of the hypertension grade.

## 4. Discussion

Despite the similarity between the confounding factors, the link existing between HL and hypertension was observed in the current cross-sectional inspection.

Overall, the hypertension, together with systolic hypertension and diastolic hypertension, was more frequent in the occupational noise-exposed workers. There might be an augmented HL in response to the hypertension levels. This outcome could be predicted by an earlier examination [12–14]. The stria vascularis is located in the lateral cochlear wall and is responsible for sending auditory signals from the cochlea to the central nervous system [15]. Vascular supply to the stria vascularis is derived from terminal arteries with no collateral supply. Therefore, the stria vascularis is particularly sensitive to events that compromise the vascular supply. Animal studies have shown reduced endocochlear potential and HL after an anoxic event [16, 17]. It is hypothesized that hypertension may compromise the vascular supply to the stria vascularis, thereby leading to HL [7]. In the current study, the hypertension group exhibited the highest risk of HL in comparison with the No HT group (*P*<0.05). The mechanism underlying the impact of hypertension on hearing is not clear. This relationship between HL and arterial hypertension has been the subject of investigations in recent decades, but the findings are inconsistent [18–21].

With aging there is a substantial increase in the number of chronic diseases [22–24]. Systemic arterial hypertension, together with HL, is common in the elderly [25]. Different investigations provide justification that sensorineural HL occurs with aging and is associated with microcirculatory inadequacy, secondary to vascular occlusion from emboli, haemorrhage, or vasospasm. Moreover, sensorineural HL is associated with hyperviscosity or microangiopathy stemming from diabetes or hypertension, and the latter condition gives rise to sensorineural HL [26–28]. We investigated the higher age range as an independent risk factor for hypertension and HL [4]. With the specific goal for the removal of confounding factors, such as age, we carried out binary logistic regression analysis. We showed that hypertension is a risk factor for HL in noise-exposed workers (*P *<0.05). As revealed by the findings, the impact of grade 1 hypertension, grades 2 and 3 hypertension, and isolated systolic hypertension on hearing impairment was quite evident (*P*<0.05). Moreover, the subjects with noise exposure were at substantially higher risk for HL in comparison with the No HL group (*P*<0.05).

In comparison with the No HL group, the subjects in the HL group and gender were at higher risk (*P*<0.05). The mean arterial pressure was higher in males compared to females [29, 30]. Pulsatile pressure primarily predominates in females, especially the elderly, as a consequence of short stature [31, 32]. Male gender was confirmed to be an independent risk factor for HL (*P*<0.05).

The current research work had a number of limitations. The size of the cohort work was greater in comparison with former research. The research subjects in this case-control study were Chinese. Our study was a case-control and did not explain whether or not hearing loss was caused by high blood pressure or high blood pressure was caused by hearing loss. Based on the results of the present study, how hypertension promotes occupational noise-induced HL warrants a prospective cohort study in the future.

## 5. Conclusion

Our current investigation validates the possibility of a relationship between hypertension and HL. Patients with hypertension exhibit a substantial rise in HL in comparison with patients without hypertension. The rise in HL was significant in patients with grade 2 hypertension. Efficient and practicable measures for decreasing the risk of hypertension and work-related noise exposure will decrease the risk of HL. The link existing between the augmented hearing limit and hypertension in this study indicates the importance of collaboration between otorhinolaryngologists, audiologists, and other medical experts dealing with the complications arising from hypertension.

## Figures and Tables

**Table 1:** Grading of hypertension DBP and SBP.

Blood pressure (mm Hg)	Grade 1 (Stage 1 hypertension)	Grade 2 (Stage 2 hypertension)	Grade 3 (Stage 3 hypertension)	Grade 4 (isolated systolic hypertension)
SBP	140-159	160-179	≥180	≥140
DBP	90-99	100-109	≥110	<90

**Table 2:** Demographic characteristics of hypertension of the subjects with occupational noise exposure.

Variable	No HT	Grade 1 HT	Grade 2 HT	Grade 3 HT	Grade 4 HT	p value
No. of workers	240165	12723	1990	585	12303	
Age (yr)	33.30(9.19)	39.91(9.79)	42.79(9.36)	43.55(8.70)	38.35(11.12)	<0.01
Mean (SD)						
Gender (No.)						
Male	181497	10829	1665	503	10184	<0.01
Female	58668	1894	325	82	2119	<0.01
Working age (yr)	9.65(8.55)	14.03(10.08)	15.80(10.43)	16.06(10.51)	12.68(10.26)	<0.01
Mean (SD)						
Work age of noise exposure (yr)	5.21(6.37)	7.93(8.07)	8.39(8.41)	8.58(8.49)	7.20(7.73)	<0.01
Mean (SD)						
No.of Hearing loss workers (%)	100247(41.74)	5860(46.06)	997(50.10)	277(47.35)	6089(49.49)	<0.01

**Table 3:** Binary logistic regression analysis of hearing loss and hypertension adjusted for age, gender, and SBP (OR: odds ratio; CI: confidence interval).

Hearing loss (vs No HL)		*OR (95%CI)*	*P*
	Age	1.050(1.050-1.051)	<0.01
	Gender		
	Male	1.438(1.426-1.461)	<0.01
	Female		
	SBP	1.046(1.032-1.060)	<0.01

**Table 4:** Binary logistic regression analysis of hearing loss and hypertension adjusted for age, gender, and DBP (OR: odds ratio; CI: confidence interval).

Hearing loss (vs No HL)		*OR (95%CI)*	*P*
	Age	1.051(1.050-1.052)	<0.01
	Gender		
	Male	1.442(1.422-1.463)	<0.01
	Female		
	DBP	1.003(1.001-1.005)	<0.01

## Data Availability

The population data used to support the findings of this study have not been made available because the Chinese law did not allow the sharing of basic information about individuals.
